# Co‐Producing Patient‐Reported Experience Measures With People With Intellectual Disability to Improve Healthcare Quality and Outcomes: The ‘Listen to Me’ Project Protocol

**DOI:** 10.1111/hex.70418

**Published:** 2025-08-29

**Authors:** Reema Harrison, Bronwyn Newman, Beth Catlett, Laurel Mimmo, Ling Wu, Maya Tokutake, Karen Phillips, Matthew Van Hoek, Debbie Van Hoek, Elizabeth Manias, Pandora Patterson, Dalal Dawood Baumgartner, Kim Bowen, Julian Trollor, Sue Woolfenden, Iva Strnadova, Johanna Westbrook, Rebecca Mitchell, Angela Dew, Patrick Olivier, Virginia Mumford, Tim Badgery‐Parker, Louise Ellis, Sophie Rodier, Tracey Szanto, Jacqueline Small, Corey Adams, Michelle Templeton, Jane Evans, Nadine A. Hackl, Kate Churruca, Anne Marie Hadley

**Affiliations:** ^1^ Australian Institute of Health Innovation Macquarie University Sydney Australia; ^2^ Faculty of Information Technology Monash University Victoria Australia; ^3^ School of Nursing and Midwifery Monash University Victoria Australia; ^4^ Community First Step Fairfield Australia; ^5^ Satb2Connect Westfield Australia; ^6^ National Centre of Intellectual Disability Health University of New South Wales Kensington Australia; ^7^ Sydney Local Health District Camperdown Australia; ^8^ School of Education University of New South Wales Kensington Australia; ^9^ Institute for Health Transformation, Faculty of Health Deakin University Victoria Australia; ^10^ Safer Care Victoria Victoria Australia; ^11^ New South Wales Agency for Clinical Innovation St Leonards Australia; ^12^ Centre for Developmental and Disability Health, Monash Health Doveton Australia; ^13^ St Vincent's Health Australia East Sydney Australia; ^14^ Bureau of Health Information St Leonards Australia; ^15^ New South Wales Ministry of Health St Leonards Australia

**Keywords:** codesign, consumer led research, healthcare access, healthcare outcomes, healthcare equity, intellectual disability, patient experience, study protocol

## Abstract

**Introduction:**

Intellectual disability, defined by significant limitations in both intellectual functioning and adaptive behaviour with onset during the developmental period, affects an estimated 2% (108 million) of people worldwide. People with intellectual disability experience major health inequity, poor health outcomes and premature deaths, with mortality rates that are 7–12 times higher than the general population. Patient‐reported experience measures (PREMs) are used worldwide to target improvements in healthcare delivery. Yet, systematic review evidence confirms that people with intellectual disability are excluded from PREMs due to a lack of suitable measurement instruments and supports. To improve healthcare quality and outcomes, people with intellectual disability and their supporters, and academic and clinician researchers will together Coproduce PREMs for, and with, this population apply the PREM in hospitals and use the data to inform local quality improvement projects.

**Methods and Analysis:**

Our 3‐year project employs a multi‐method design using coproduction, underpinned by an implementation science framework. We will coproduce PREMs that will be applied to improve healthcare quality and outcomes for people with intellectual disability whilst engaging in co‐research with people with lived experience of having an intellectual disability and their carers, clinicians and academic researchers. Study 1 will coproduce PREMs for use by people with intellectual disability to report their experiences of inpatient hospital care. Study 2 will use the co‐produced PREMs to capture the experiences of people with intellectual disability as inpatients in Australian hospitals; determine PREMs reliability and validity; and costs associated with use. Study 3 will develop the required capability to translate our PREMs into hospitals across Australia. Study 4 will apply PREMs data via continuous quality improvement in partnering hospitals to reduce preventable healthcare associated harm, hospitalisation, and prolonged length of stay experienced by people with intellectual disability. Study 5 will provide a process evaluation of our co‐research approach. Quantitative and qualitative analysis will be undertaken, and results will be examined against the project aims.

**Ethics and Dissemination:**

Ethical approval has been obtained (520241735259588; X24‐0366; 115771). This study is being conducted with partner health agencies and services nationally in Australia to support achievement of the research aims and translation of findings into practice. Targeted outputs with research dissemination will be guided by, and in collaboration with, the project Consumer Leadership Group, consumer and health system stakeholders, with governance from the Project Steering Group.

**Patient or Public Contribution:**

The Listen to Me project has been designed and planned to ensure the involvement of people with a broad range of abilities, including those with profound intellectual disability who will be able to contribute along with their support person/s. The governance structure of Listen to Me is innovative and inclusive, with consumers providing leadership across all elements of the project. This is co‐research conceptualised and conducted with a Consumer Leadership Group (CLG) comprising eight people with lived experiences of intellectual disability; two of whom have an intellectual disability and six of whom are parents or siblings who support family members with intellectual disability. The CLG were involved in the design of the research proposal, reviewing and contributing to the ethics protocols, and the writing of this protocol as authors. The CLG will direct and contribute to all aspects of the research as part of the research team. An easy read summary of this protocol is provided in Supplementary file 1.

## 1 Introduction

Intellectual disability, defined by significant limitations in both intellectual functioning and adaptive behaviour with onset during the developmental period [1], is estimated to affect 108 million people (2%) worldwide [2]. People with intellectual disability have significantly higher levels of physical and mental health needs than the general population, which contribute to high healthcare utilisation from a young age, with 1 in 7 children in hospital having an intellectual disability [3, 4].

People with intellectual disability experience major health inequity, poor health outcomes and premature deaths, with mortality rates that are 7–12 times higher than the general population [5, 6]. At a population level, key drivers of this inequity are unrelated to the aetiology of the disability itself. Instead, the genesis stems from poor healthcare quality, and the compounding effect of socio‐cultural determinants of health. A key contributor to disparate outcomes is inequitable healthcare quality [7]. This has been demonstrated by recent studies into the poor quality of care experienced by people with intellectual disability throughout health systems globally [4, 8, 9, 10, 11, 12, 13, 14]. People with intellectual disability are hospitalised for acute conditions that should not require hospital‐based care 5–8 times more often than the general population [12]. Once in hospital, they have a 30% higher rate of preventable healthcare‐associated harm than the general population [3, 11].

Current evidence demonstrates that there is a greater propensity for errors to occur in the health care of people with intellectual disability, and that concerning changes in physical and mental health are overlooked because they are misattributed to the intellectual disability [3, 9, 11]. Poor health service quality increases the risk of deterioration in health requiring prolonged length of stay (LOS) in hospital, potentially preventable hospitalisation via readmission, and preventable emergency department (ED) presentations for people with intellectual disability. So stark is the healthcare quality gap that people *with intellectual disability have been described as “subject to systemic neglect”* in healthcare systems [15].

The importance of listening to, and acting upon, patient experiences for improving healthcare quality is well‐established. For example, an analysis of > 7000 patients in Australian hospitals highlighted the significant association between patients' poor experiences of hospital care and the presence of healthcare‐associated harm [16, 17, 18]. Patient Reported Experience Measures (PREMs), typically conducted via survey, are now routinely collected and used to drive continuous healthcare improvement internationally [16, 19].

Despite the critical contribution of patient‐reported experience data identifying and addressing issues of healthcare quality, a 2022 literature review reported that no PREMs instruments have been developed for, and with, people with intellectual disability [20]. Importantly, the review highlighted the need for PREMs data collection instruments, along with suitable approaches for collecting these data. PREMs used by regional and national health organisations and agencies, such as the United Kingdom (UK) National Health Service, the Australian Commission on Safety and Quality in Health Care and the United States (US) Agency for Healthcare Research and Quality often contain as many as 50–80 survey items [20]. Beyond the number of items, current PREMs frequently include complex written information, with items that require recall of specific scenarios, words with multiple meanings, and questions with multiple elements [20]. Current tools reply on the use of proxy for completion; this lacks alignment with the United Nations Convention on the Rights of Persons with Disabilities (CRPD) which highlights that people with intellectual disability have a right to voice their own opinions directly [21]. The review further identified that although qualitative data collection has successfully elicited experiences from small numbers of people with intellectual disability, surveys used for large scale data collection are not available in modes such as Easy Read to facilitate their access and use by people with intellectual disability [20].

The urgent need for suitable PREMs for use by people with intellectual disability to report and seek to improve their healthcare experiences led to the establishment of a research programme entitled the Listen to Me project. Through coproduction and co‐research, the Listen to Me project will create and apply PREMs with people with intellectual disability in Australian hospitals.

## 2 Aims

The overall goal of this study is to develop and use patient‐reported information to improve healthcare quality among people with intellectual disability in Australian hospitals. This goal will be achieved by the completion of five studies corresponding to the following five aims.

1. Coproduce PREMs with people with intellectual disability regarding hospital care.
2. Capture the experiences of people with intellectual disability in Australian hospitals using the Listen to Me PREM and determine the PREM's reliability, validity, and associated costs.
3. Develop capacity building resources for health care providers and consumers to translate the Listen to Me PREMs into routine practice in hospitals.
4. Apply PREMs data to improve care quality among people with intellectual disability in hospitals.
5. Evaluate the perceived suitability and impacts of co‐research approaches used in the Listen to Me project.

## 3 Methods

### 3.1 Research Design

A mixed‐methods sequential design will be employed comprising Coproduction, survey and interview data collection with patients, families and health service staff and evidence synthesis. The overall completion of the project is expected to take 3 years (2023–2026).

### 3.2 Project Governance

Project governance is provided by a Project Steering Group (PSG) comprising of 10 members that include people with lived experience of having and supporting others with intellectual disability along with representives from health systems and services organisations from a range of locations across Australia. The PSG members are external to the project and the role of the group is to provide oversight of project processes through twice yearly meetings. The inclusion of a PSG ensures external oversight through an independent mechanism and supports knowledge creation in transdisciplinary research [22]. A Consumer Leadership Group (CLG) is also established, which is an internal group of eight individuals who have lived experience of having or supporting someone with intellectual disability accessing hospital care. CLG members are co‐researchers who have designed Listen to Me, acquired funding as investigators and direct the research process. CLG members have trained in research members with clinicians and academics ahead of commencing the Listen to Me project. The CLG sends two members to each PSG meeting to provide a report on the function and work of this group throughout the project.

## 4 Study 1: Coproduction of Patient‐Reported Experience Measures

### 4.1 Design

Study 1 utilises Coproduction methodology which refers to “*the direct contribution of patients, service users and/or family members to the health or wellbeing service from which they (or their family members) benefit”* [23]. Co‐producing PREMs with people with intellectual disability will ensure the tool addresses aspects of patient experience that matter to people with intellectual disability and in ways that are readily understood by, and suitable to use with, this population. Using Coproduction [24], a group of people with lived experience of intellectual disability, academics and health system staff will collaborate to produce a digitised PREM survey and supporting resources for use by people aged 6 years and older with intellectual disability. We will focus on this age group because existing parent experience tools are in development and many children aged < 5 years may have a diagnosis of Global Developmental Delay rather than intellectual disability. Consensus on the final survey items included in the PREM will be determined via a modified‐Delphi study.

### 4.2 Sample

A Coproduction group will develop an initial prototype PREM. The group will comprise of the eight members of the Consumer Leadership Group, with invited input and guidance via a series of workshops throughout the process from members of the wider project team, including human computer interaction specialists, multi‐disciplinary academics spanning special education, intellectual disability health, and health system and services. Beyond the initial prototype PREM, user testing and refinement will occur with up to 50 people with intellectual disability recruited via project networks and social media to ensure a broad range of abilities and perspectives are considered. Up to 100 further people who have intellectual disability or provide support or care for someone with intellectual disability, will be invited to take part in the Delphi survey study.

### 4.3 Recruitment

Potential participants for user testing and the Delphi components of the study will be sent accessible study information via our professional networks and social media. Those wishing to take part will be provided with the opportunity to discuss their support needs with the research team.

### 4.4 Procedure

The study will be undertaken in three parts: Coproduction workshops, user testing and a modified two‐round Delphi study. Appropriate methods for gathering consent will be determined by the research team through discussion with individuals and their support persons. Written consent will be gathered from those for whom this method is suitable on paper‐based form that are stored separately from the data. Consent will be gained using accessible study information with appropriate language or cognitive support provided tailored to suit individual needs and preferences. Consent will further be reconfirmed throughout the process by the researchers verbally. These informed consent procedures will be followed throughout the research.

#### 4.4.1 Coproduction Workshops

Coproduction will take place via in‐person or online workshops conducted approximately once per month over 6 months, totalling 12–18 h [25]. Visual arts‐based communication strategies, such as drawings, photos, symbols, and video‐clips, will facilitate coproduction with people who have a range of communication preferences. Members will first review the currently available PREMs from our literature review [20] and determine the scope of adaptation required. Members will then systematically adapt the question phrasing, language and modality for greater accessibility whilst seeking to maintain the fidelity of the survey questions. Meeting timing, breaks, location, and duration will be agreed with members based on their needs and circumstances. Communication with Coproduction members between meetings will enable us to identify and respond to risks, queries, and emerging support needs.

#### 4.4.2 User Testing

The initial prototype PREM resulting from the Coproduction workshop will be subject to user testing with up to 50 people who have intellectual disability to explore their suitability, acceptability and feasibility of completion among people with a range of different abilities. The sample will be purposively recruited from a range of geographical and service delivery contexts, including people who live in metropolitan, regional and rural areas accessing a range of hospital services throughout Australia. Each participant (with support person optional) will be asked to complete brief verbal feedback, via phone, online or in‐person based on their preference, on the perceived value of the PREM, ease of completion, time taken to complete and level of support required using think‐a‐loud interview methods [26]. Usability testing data will be used to make refinements to the PREM before large‐scale administration in Study 2.

#### 4.4.3 Delphi Study

The Delphi study will be offered in two modes to facilitate the involvement of people who do and who do not require supported completion. Participants who do not require supported completion (including healthcare providers and carers for people with intellectual disability) will be sent an email including an invitation to participate in the Delphi study along with an online link through which the Delphi survey can be accessed and submitted. Two rounds of surveying will take place. The PREM survey questions will be compiled into a survey and will be sent to participants for the first round of Delphi to vote on their relevance and clarity. First round results will be used to refine the PREM items, which will be sent out for a further round of voting. Results received from the second round will be analysed to determine items with the ≥ 70% consensus to be retained [27]. For participant who require supported completion, this will be provided using an accessible Delphi study. A topic guide will incorporate the Delphi questions in a structured interview process that will be administered one‐to‐one or in small groups online or in person at locations convenient to participants. Incorporation of the Delphi study in the project design enables diverse expert stakeholder perspectives to be considered in the PREM content and design. Stakeholders include people with intellectual disability, health care workers, disability workers and policy makers. Changes to the PREM suggested in the Delphi survey can be considered in detail through the lens of the CLG's lived experience, as the CLG members comprise the core panel members in the Delphi process. The outcome of study 1 will be a set PREM suitable for field‐testing/psychometric validation.

## 5 Study 2: Measurement of Patient Experiences in Australian Hospitals

### 5.1 Research Design

Cross‐sectional survey.

### 5.2 Setting

Five health service organisations comprising > 20 hospitals that collectively see approximately 6000 young people (6–15 years) and > 8000 adults (16 years and older) with intellectual disability annually. Participating hospitals are distributed across all 8 Australian states and territories, across metropolitan, regional and rural settings.

### 5.3 Sample

A minimum sample of 580 people aged ≥ 16 and 730 people aged 6–15 years is being sought, which is sufficient to detect a small effect size (*d* < 0.2 for continuous outcomes; OR 1.2 for binary outcomes) with 80% power (*α* = 0.05) and provide adequate power to explore interactions and subgroup analyses (OR 1.7 for interaction effect with binary outcomes).

### 5.4 Recruitment

The five service partner organisations collaborating in the research will support the project team to recruit people with intellectual disability to complete the PREM at each of the sites. We will use two approaches to identify eligible participants by: (1) identifying people who have an intellectual disability that attended each service in the past 24 months, (2) advertising the study through public areas of the participating services, and by attending these in person. People who have an intellectual disability that attended each service in the past 24 months will be identified by local clinical teams. In clinics that see a large number of people with intellectual disability will distribute the outpatient Listen to Me PREM survey link via their usual email and text‐based communications. To consider participation, the research team will provide potential participants and their supporters with accessible study materials in a range of formats.

### 5.5 Procedure

Consent will be gained via accessible consent forms completed with appropriate language or cognitive support and confirmed at the time of PREM completion. People with intellectual disability will be asked to complete the PREM via a weblink or using a paper‐based version, if requested. Respondents (or their proxy) will be asked whether they consent to have their responses to the PREM linked to hospital admission records for future analysis to examine PREM scores in relation to hospitalisation outcomes of prolonged LOS (determined as longer than the national average of all patients having that procedure), and healthcare‐associated complications (HAC) (e.g., hospital‐acquired infections); secondary outcomes of preventable hospitalisations: readmission within 28 days, unplanned admissions, and emergency department (ED) representation within 72 h. Qualitative interviews will be conducted with 5–10 staff and consumers at each site to understand the implementation process, requirements and needs of the services to use the Listen to Me PREMs.

### 5.6 Analysis

Frequencies and descriptive statistics will be reported for PREM data using SAS (version 9.4) software along with inter‐comparisons by characteristics such as age group and hospital. Cronbach's coefficient alpha will be used as to determine internal consistency reliability of the PREM scale or any subscales. Construct validity will be determined using Exploratory and Confirmatory Factor Analysis techniques. The NSW Bureau of Health Information (BHI) and eHealth (Victoria) will provide comparator PREM datasets matched by age and sex to determine the concurrent validity of the PREMs; the extent to which our PREM findings correlate with data produced by current inpatient hospital PREMs. Activity‐based costing will measure the cost of collecting PREMs from people with intellectual disability. An activity map will be developed to identify critical stages in the data collection process to inform our data collection strategy. We will measure PREM completion times and assess the role of facilitators in PREM completion using qualitative interviews with 5–10 staff at each site. We will determine the costs and scalability of the new PREMs service using sensitivity analyses.

## 6 Study 3: Developing Capacity Building Resources for the Translation of PREMs to Practice

### 6.1 Research Design

Framework synthesis [28] and Coproduction.

### 6.2 Data Sources

Qualitative data from Studies 1 and 2 about support required for consumers to complete PREMs and for staff to administer the PREM, including capacity and capability gaps and how existing resources were leveraged during data collection in Study 2, will be synthesised with our systematic review evidence of PREMs approaches with people with intellectual disability [20].

### 6.3 Co‐production Group Members

A multidisciplinary team comprising the eight CLG members, clinicians from partnering health services, and academics with expertise in patient reported measurement and intellectual disability health.

### 6.4 Procedure

Two stages of research will enable the capability gaps to be determined (evidence synthesis) and then supporting resources to be produced (coproduction).

### 6.5 Evidence Synthesis

**Figure 1:**
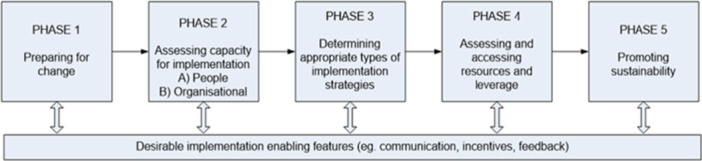
Translational model for implementation success.

Our multidisciplinary team will synthesise the multi‐source data to distil the capability support required for the implementation of PREMs relative to an evidence‐based translational model (Figure 1) [29]. Data will be indexed to synthesise key information relevant to the elements of the model, considering different settings and sub‐populations [28]. For example, support requirements to utilise the PREMs in inpatient and outpatient settings, in metropolitan, regional and rural contexts, in mental health, emergency, maternity or other speciality areas. Easy read summaries of the key information from each data source will be produced by our trained team to support the contributions of people with intellectual disability in this process. The purpose and scope of capability resources will be then agreed.

### 6.6 Co‐Producing Resources

Resulting evidence will be applied by the coproduction group to develop three packages of written and visual resources that can be utilised in conjunction as required to equip: 1. people with intellectual disability and their supporters to know of and complete PREMs, 2. health services with the capability to apply the PREMs and 3. state‐wide survey teams to integrate the Listen to Me PREMs and report these data to drive change at‐scale Using visual or arts‐based methods as appropriate (Hewitt et al.), we will review the evidence to identify the types of capability support needed. We will collaborate through a series of online or in‐person workshops facilitated by an external design agency, along with asynchronous prototyping to produce the written and visual resources that are determined as necessary for the target groups of end users. During this process, we will consult with stakeholders including people with lived experience of having or supporting someone with intellectual disability in hospital care beyond the CLG, staff from the partner services and those in state and federal health agencies.

## 7 Study 4: Applying PREM Data to Improve Care Quality Among People With Intellectual Disability in Hospitals

### 7.1 Research Design

Cross‐sectional continuous quality improvement projects.

### 7.2 Setting

Five health service organisations comprising > 20 hospitals that collectively see approximately 6000 young people (6–15 years) and > 8000 adults (16 years and older) with intellectual disability annually.

### 7.3 Procedure

PREM results from Study 2 will be provided to the participating health service organisations to inform targeted continuous quality improvement (QI) activities in each service. Local teams will devise the QI activities in each service with identified aims to improve one or more of the following care outcomes for people with intellectual disability: reduce preventable healthcare associated complications, potentially preventable hospitalisation or prolonged LOS. QI projects are likely to vary between different services but may include initiatives to identify deteriorating patients, errors in care or unmet health needs. Research team members experienced in QI, together with the CLG, will facilitate the QI projects as mentors for clinicians in each service.

### 7.4 Analysis

The Model for Improvement [30] will be employed across the project lifecycle, with evaluative data being produced using a range of QI tools iteratively (e.g., run charts, scatter plots and statistical analysis) to demonstrate the impacts of the QI activity on one or more of the four primary outcomes of interest [31].

## 8 Study 5: Process Evaluation of Co‐Research Approach

### 8.1 Research Design

Mixed method comprising document analysis and semi‐structured interview.

### 8.2 Sample

All Listen to Me Project meeting documentation, including agendas minutes, recordings and related communication. All project team members including the PSG, CLG, wider investigatory team and employed project staff will be invited to take part in interviews by an independent researcher. Project members will be informed that participation in the evaluation is voluntary, optional and confidential.

### 8.3 Recruitment

All project team members will be invited to take part in evaluation interviews. Team members will be made aware of this opportunity at project meetings and via email invitation with supporting study information and consent form.

### 8.4 Procedure

Interviews with the Listen to Me project team will be conducted in‐person, online or via telephone once each year over the 3 years of the project by an external researcher. Co‐research approaches, governance, inclusive practices, meeting structure, project practices, challenges and opportunities will be examined via a semi‐structured schedule. Interviews will be recorded and transcribed for analysis.

### 8.5 Analysis

Framework analysis [32] using key characteristics of inclusive research and governance [33, 34, 35] to distil key strategies and principles to inform practices in Listen to Me and future projects.

## 9 Expected Outcomes

By conducting coproduction with people with intellectual disability, their supporters and clinicians, this study will provide a novel PREM that will aid understanding of the hospital experiences of people with intellectual disability from their viewpoint. Specifically, the following new insights and outputs will result:

- Co‐produced, digitally enabled and validated PREM for people aged ≥ 6 years that facilitates expression of the hospital experiences of adults, young people and school‐aged children with intellectual disability by using visual arts‐based communication strategies.
- PREM data from people with intellectual disability in Australia and evidence of associations between experiences of hospitalisation and care outcomes, providing knowledge of targets for service improvement.
- Suite of resources for capability development to ensure that research outcomes can be implemented and translated into policy and practice.
- QI activities that improve care outcomes for people with intellectual disability in hospital. Health system partners will support knowledge translation of QI activities that improve care across state health systems in Australia.
- Advancement of evidence about methods for health service co‐research between people with intellectual disability, their supporters, clinicians and multidisciplinary academics.

The research community of academics, consumers and clinicians will together build greater capacity and capability to undertake translational research with people with intellectual disability and their supporters. Key mechanisms for this are peer mentorship in translational research practice between consumer, clinical, academic and system staff. The project will also provide evidence about how to translate knowledge into action in health services research by creating and embedding implementation support for the use of PREMs with people with intellectual disability.

## 10 Patient or Public Involvement

Consumers have been involved in the design and planning of this study project from inception, with the primary mechanism to facilitate ongoing involvement from a diverse range of individuals via the CLG. Consumer involvement will continue to occur at every stage of the research, including governance, and all project documentation will be provided in formats accessible to consumer participants. Continued mentorship between clinicians, academics and consumers will support involvement in the ongoing conceptualisation, development, planned translation and implementation of the research. This model of working embeds lived experience into the day‐to‐day conduct of the Listen to Me project.

## 11 Ethics, Data Storage and Protection

The Human Research Ethics Committee at Macquarie University, Monash Health and Sydney Local Health District approved this study (Project ID 520241735259588; X24‐0366; 115771). We will obtain written informed consent from those who complete PREMs and interview research. We will ensure participants' confidentiality and anonymity throughout the study, including during data gathering, management, analysis and reporting processes. All data from this study will be securely managed and protected, and only the researchers involved in the study can access the data. We will store electronic data, such as audio recordings and survey responses, on the Macquarie University OneDrive, which is a secure, password‐protected platform that requires two‐factor authentication. Participant consent forms will be stored separately to their PREM and health data, remaining in the hospital sites. It is not anticipated that the data will be used for any other purpose, and a new ethics application will be made should there be a proposal to use the data for another purpose. The data will be destroyed per ethical requirements 7 years after the project's completion.

## 12 Projected Outputs and Dissemination Plans

There is an international imperative to listen to, and act upon, the wishes, needs and preferences of people with intellectual disability as a human right and redress inequity in health outcomes [21]. Our co‐produced PREM provides a much needed mechanism for people with intellectual disability to communicate the views and needs; as such, the PREM along with supporting resources and resulting data about experiences must be disseminated widely and in accessible formats. The study findings will be disseminated using a range of formats and outlets to ensure that all stakeholder groups with interest in the project and its outcomes are able to access and interact with the findings. Priority will be given to providing information to participants and interested consumers in accessible formats (e.g., Easy Read or video summary). The consumer‐led governance structure of this project will ensure that consumers actively inform the plan for research dissemination throughout the project. The CLG members will also have opportunities to inform and contribute to dissemination activities such as academic publications, presentations, and developing accessible summaries to share findings. Targeted dissemination will seek to inform the National Safety and Quality in Healthcare Standards in Australia along with federal and statewide patient experience measurement approaches.

## 13 Anticipated Strengths and Challenges

Our history of collaborating in co‐research between consumers, clinicians and academics is a core strength of this study, along with the employment of people with intellectual disability as members of the research team. By adopting a staged approach, employing coproduction with people who have a range of experiences and types of intellectual disability, along with expansive consultation and user testing, our PREMs will meet the needs of people with a range of abilities and communication styles and produce data that can be completed and used by stakeholders to understand and improve experiences of care. Substantial funding allocated to remuneration of consumers is a further strength of the project which will enable time‐consuming and complex work to be undertaken with rich consumer input. Our experience as a team in researching health care experiences among people with intellectual disability equips our researchers with the skills and awareness to navigate this study appropriately and provide the required support to achieve the research aims. Despite these strengths, the research may be constrained by the challenges in readily identifying people with intellectual disability in hospitals, leading to slow recruitment and a long time taken to achieve the desired sample size and representativeness. Working in partnership with multiple health services comprising > 20 hospitals that see several thousand people with intellectual disability each year provide support for promoting and attracting participants to engage with the research. Although there is rich and broad engagement with people who have a range of abilities and communication styles during coproduction, there are likely to be challenges implementing the Listen to Me PREM in real‐world healthcare settings. Healthcare staff and support persons will require the capability and capacity to ensure that people with intellectual disability gain access to the PREM to gather these data routinely. Services will need to ensure that the PREM completion and the use of these data are considered in their workflows and complement existing clinical systems to have an impact on care. Finally, it is possible that the resulting PREM will not be able to meet the needs of all people with intellectual disability given the diversity of the population, and that further refinement may be required.

## 14 Discussion

Addressing inequities in health outcomes experienced by people with intellectual disability is contingent upon having their healthcare wishes and needs listened to and acted upon [15]. Patient experience measurement and its use in hospital settings provides a mechanism for patients to provide their perspective about what is happening to them and what they need. PREMs have been used as a quality improvement mechanism leading to changes in healthcare processes and practices throughout health settings internationally [36]. Until now, people with intellectual disability have not been able to directly report their own experiences of care and have been excluded from this important quality improvement mechanism. With PREMs considered a critical tool to ensure healthcare providers know about, and work to promote, the experiences of patients in hospital settings, the Listen to Me PREM presents a critical step forward in improving the care of people with intellectual disability.

By co‐producing and applying the Listen to Me PREM, this study will provide the first PREM designed with and for completion directly by people with intellectual disability to report on their inpatient hospital experiences across hospital speciality settings, sites and health systems nationally. Our PREM will enable health service providers to attend to the rights of people with intellectual disability to have their wishes heard and acted upon and meet their obligations in providing quality care. The new knowledge generated from this use of this tool will inform healthcare providers, the public, regulators and government agencies about gaps in care provision, and identify target areas for action to ensure greater equity is achieved in the safety and quality of care provision among people with intellectual disability. Using the Listen to Me PREM further provides health services with a tool to measure and report on improvement efforts in care. Given the absence of suitable tools internationally for this community, the Listen to Me PREM may in future be evaluated for its use in health systems beyond the Australian context.

## Author Contributions

The project was conceptualised and methodology developed by all authors in developing the project idea to seek funding and who ultimately acquired the funding. Software development has been led by Ling Wu and Patrick Olivier. Data curation has been led by Bronwyn Newman, Beth Catlett and Ling Wu. Authors responsible for investigation are Reema Harrison, Bronwyn Newman, Beth Catlett, Laurel Mimmo, Maya Tokutake, Karen Phillips, Matthew Van Hoek, Debbie Van Hoek, Elizabeth Manias, Pandora Patterson, Dalal Dawood Baumgartner, Kim Bowen, Sue Woolfenden, Iva Strnadova, Rebecca Mitchell, Angela Dew, Patrick Olivier, Virginia Mumford, Louise Ellis, Tracey Szanto and Jacqueline Small. Validation and visualisation has been led by Ling Wu and Beth Catlett. Formal analysis has been led by Reema Harrison, Bronwyn Newman, Beth Catlett < Ling Wu, Tim Badgery‐Parker with supervision provided by Reema Harrison, Julian Trollor. Rebecca Mitchell and Virginia Mumford. All authors contributed to writing, reviewing and editing the manuscript.

## Conflicts of Interest

The authors declare no conflicts of interest.

## Supporting information

Helping people with intellectual disability to say what they think about health care to make it better.

## Data Availability

Data sharing is not applicable to this article as no new data were created or analyzed in this study.
