# Supplementary material for: Co‐Producing Patient‐Reported Experience Measures With People With Intellectual Disability to Improve Healthcare Quality and Outcomes: The ‘Listen to Me’ Project Protocol
Source: Health Expect. 2025 Aug 29;28(5):e70418. doi: 10.1111/hex.70418 (PMC12397070; doi:10.1111/hex.70418)
Supplement: Supplementary file 1 — Helping people with intellectual disability to say what they think about health care to make it better. [file HEX-28-e70418-s001.docx]

**Listen to Me Project**

**Helping people with intellectual disability to say what they think about health care to make it better**

| The problem we are solving | |
| --- | --- |
|  |  |
| 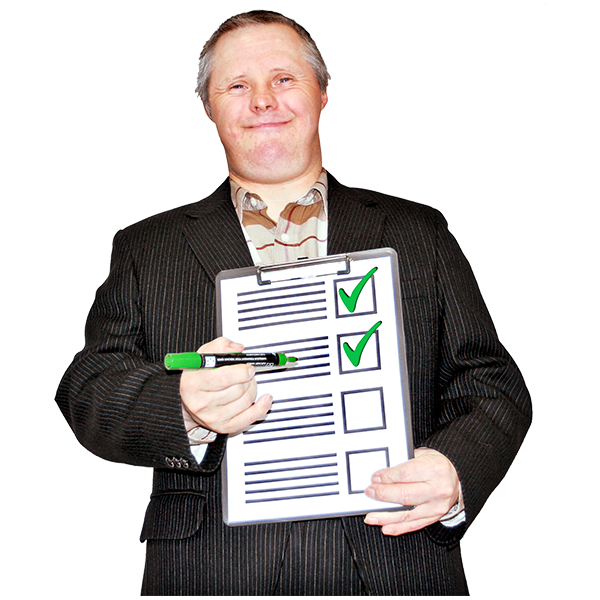 | **Patient Reported Experience Measures** or **PREMs** are surveys that ask people about their health care. |
| 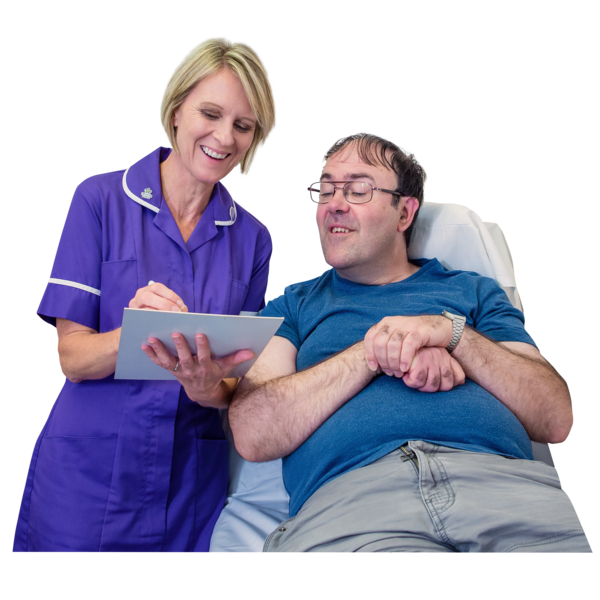 | People can use **PREMs** to say what they like and do not about their health care to make it better. |
| 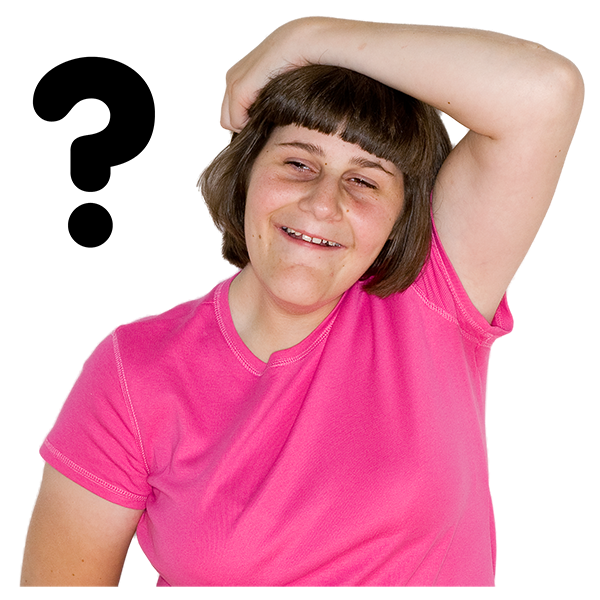 | The problem is that **PREMs** are hard for people with intellectual disability to do. |
| 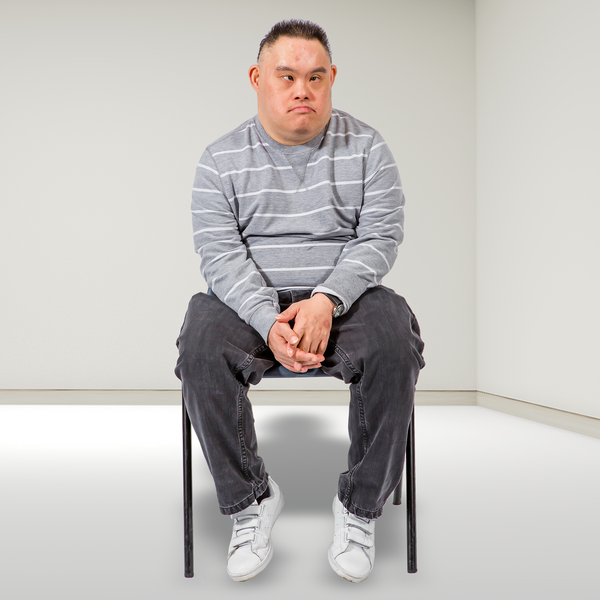 | So people with intellectual disability do not get to use **PREMs** to say what they like and do not like about their health care. |
| 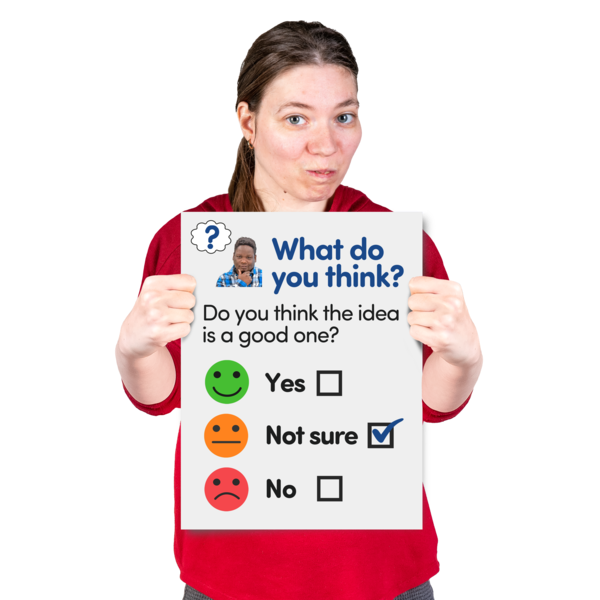 | We need to find a way people with intellectual disability can use **PREMs** like everyone else. |
| 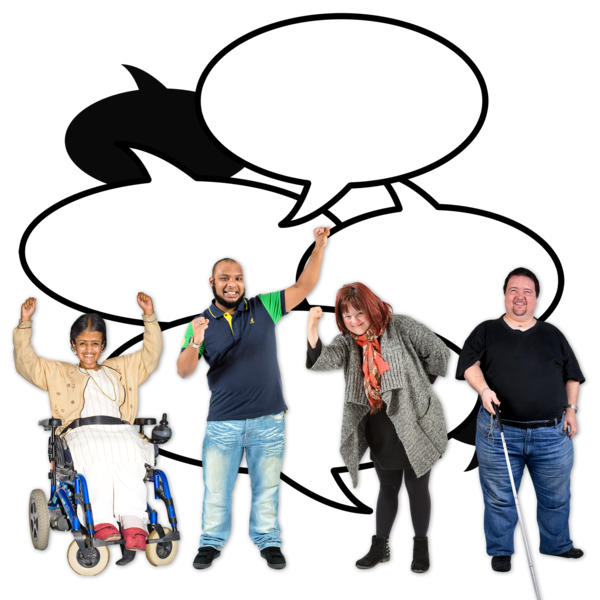 | This will make it fair and give people with intellectual disability a chance to say what they like and do not like. |
| The research project | |
| 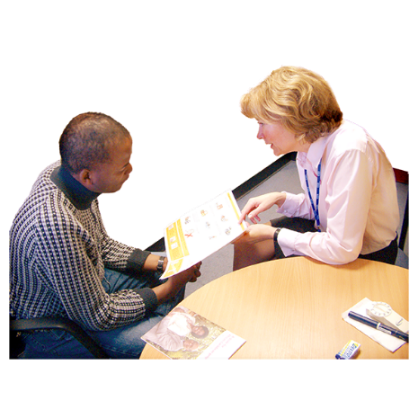 | Our project will make a **PREM** survey that people with intellectual disability can use. |
| 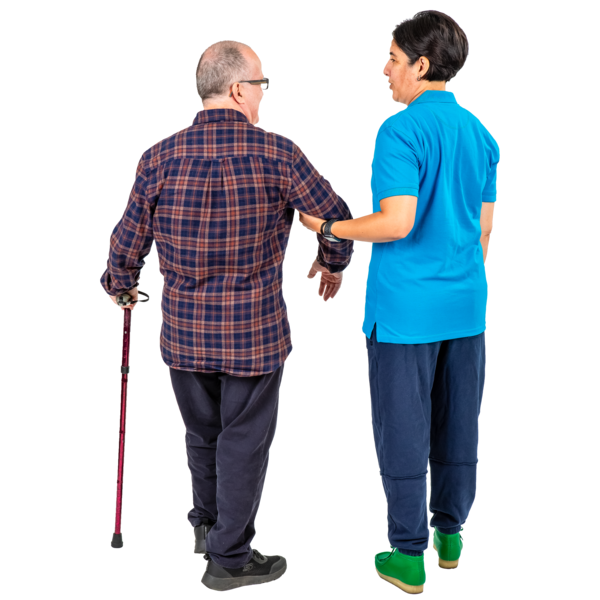 | People with intellectual disability will be able to use the **PREM** surveys to say what they like and do not like about their health care. |
| 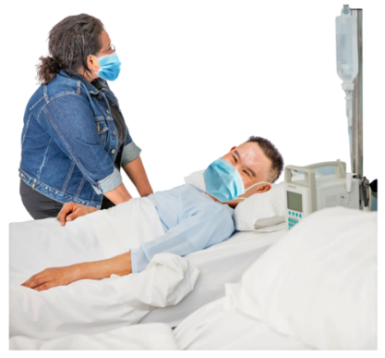 | Health care workers will use what people tell them in the PREM to make health care better for people with intellectual disability. |
| The research team | |
| 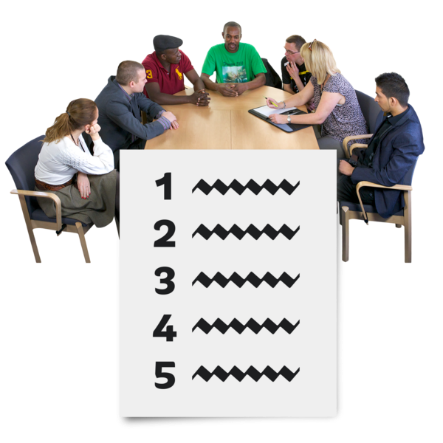 | Our **research team** members are:   - People with intellectual disability. - Family members of people with intellectual disability. - Doctors and nurses who work at hospitals. - Researchers who work at universities. |
| 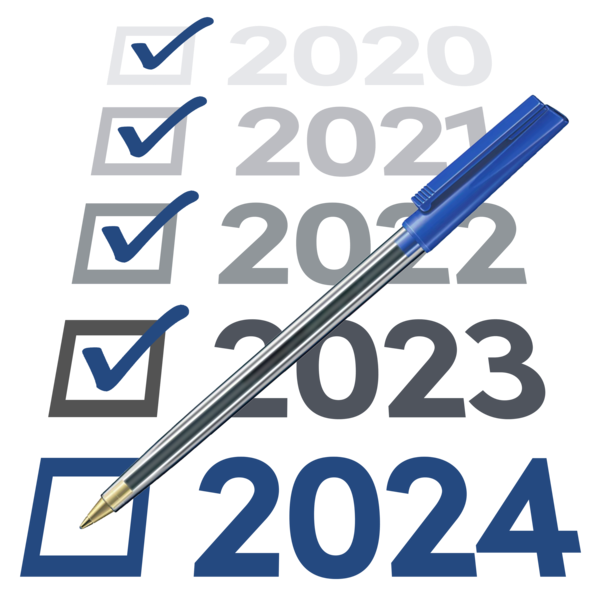 | We have worked together for more than 5 years. We have done good work. |
| 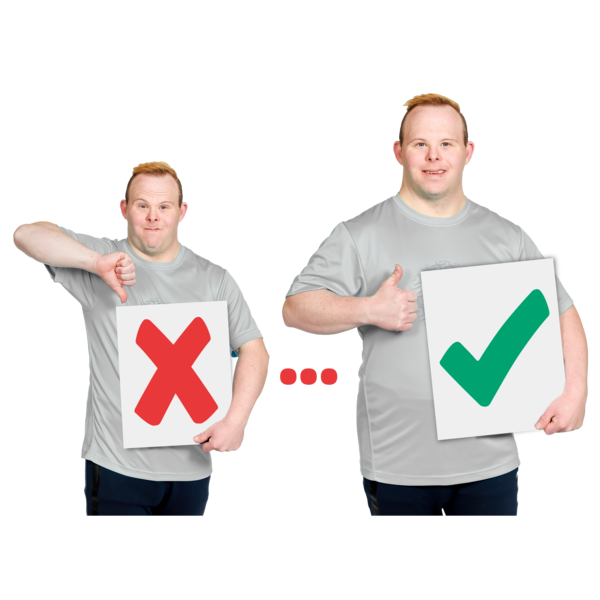 | We know that making health care better for people with intellectual disability is very important. |
| How we will do our research | |
| 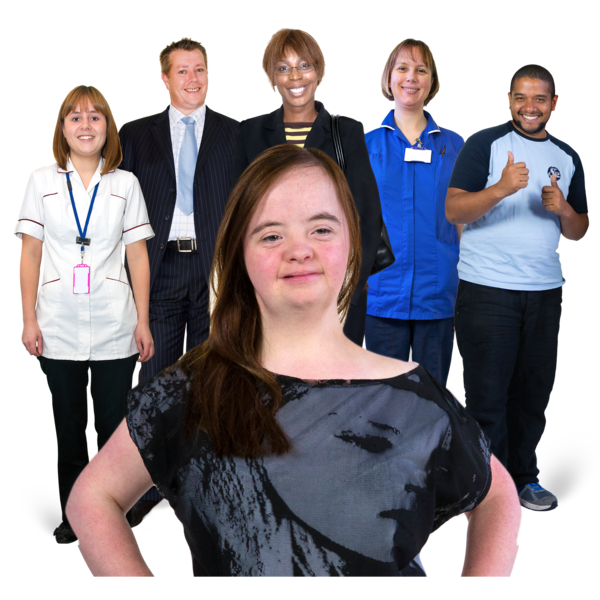 | The **research team** decides how to do the research. It does the research jobs like interviewing people. |
| 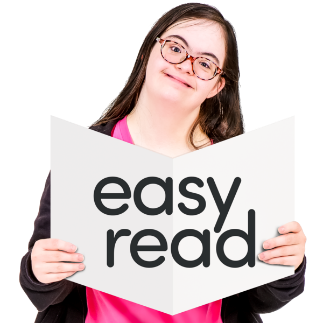 | The **research team** will explain things clearly so everyone can understand. |
| 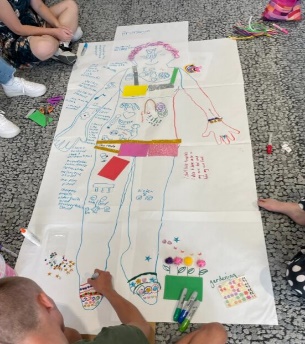 | We will use art and pictures to make it easy for people to join in our research. |
| 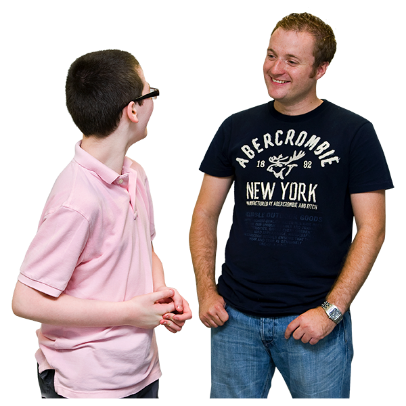 | People in our research can have a support person with them to help. |
| 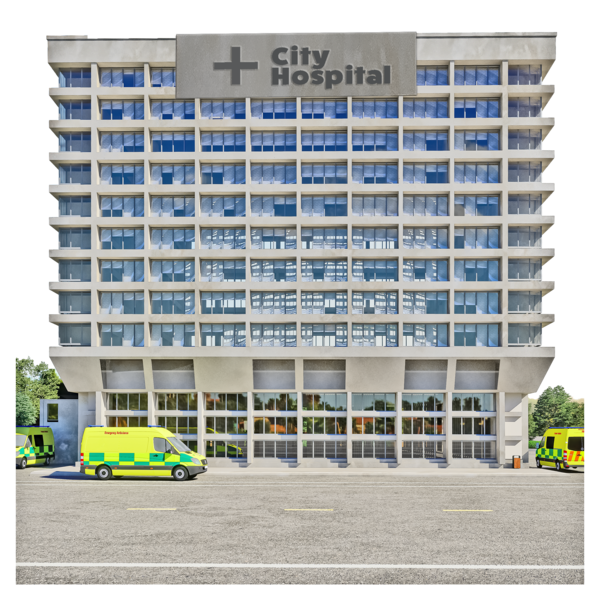 | We will get help from universities, hospitals and government departments in Australia to do the research. |
| What we will be doing | |
| 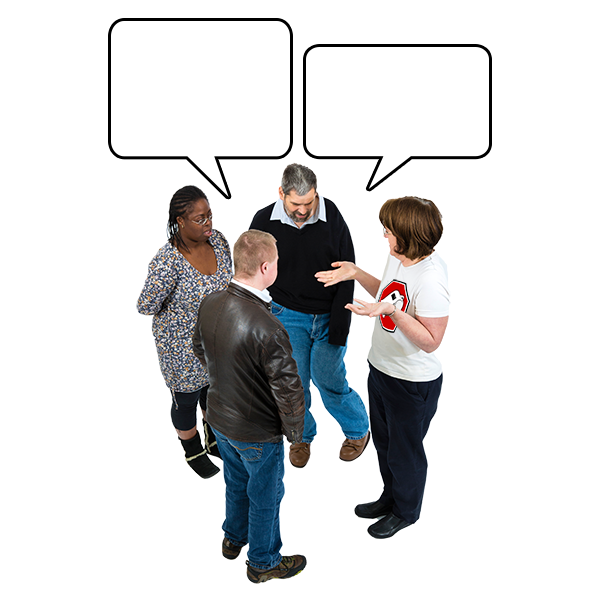 | We will ask people with intellectual disability and their carers what they like and do not like about health care. |
| 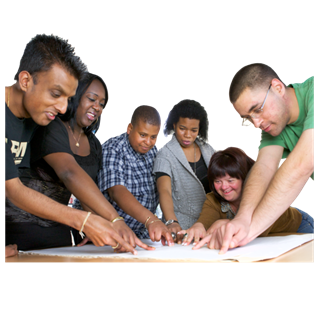 | We will find out what health care people with intellectual disability want to tell health care workers. |
| 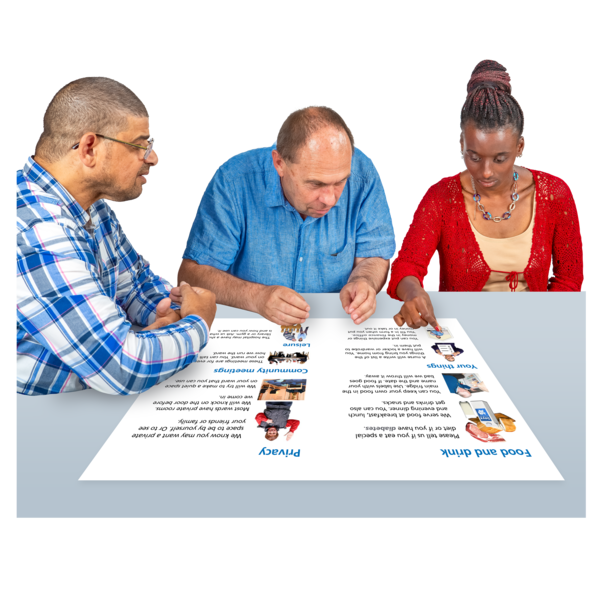 | We will make a **PREM** for and with people with intellectual disability to say what they like and do not like about their health care**.** |
| 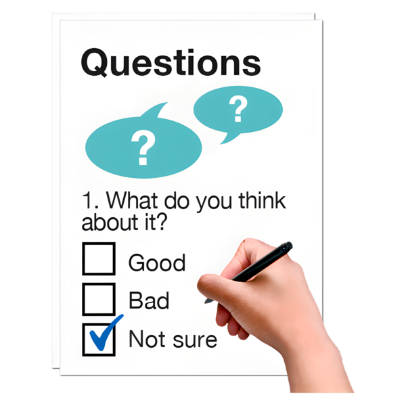 | People with intellectual disability will try using the **PREM** to say what they like and do not like. |
